# Development and Preliminary Clinical Evaluation of a Five-Item Prepubertal Risk Questionnaire for Severe Spermatogenic Impairment: A Retrospective Validation Study—Prepubertal Risk Questionnaire for Spermatogenic Impairment

**DOI:** 10.3390/diagnostics16142171

**Published:** 2026-07-11

**Authors:** Sandro La Vignera, Rosita A. Condorelli

**Affiliations:** Department of Clinical and Experimental Medicine, University of Catania, Via S. Sofia 78, 95123 Catania, Italy; rosita.condorelli@unict.it

**Keywords:** azoospermia, male infertility, prepubertal, risk questionnaire, spermatogenesis, fertility preservation, oligozoospermia, asthenozoospermia, teratozoospermia

## Abstract

**Purpose**: Severe spermatogenic impairment, including azoospermia, oligozoospermia, asthenozoospermia, and teratozoospermia, represents a major reproductive health concern, yet no validated screening tool exists for early risk stratification in prepubertal boys. We aimed to develop a five-item evidence-based questionnaire and conduct preliminary clinical evaluation to identify prepubertal boys at risk of developing severe spermatogenic impairment. **Methods**: This retrospective observational study analyzed medical records of 200 male patients aged 18 years who underwent their first semen analysis between 2014 and 2024 at the University of Catania, Italy. Prepubertal risk factor profiles were retrospectively reconstructed by two independent andrologists blinded to semen analysis results across five evidence-based domains: genetic anomalies (0–5 points), cryptorchidism (0–6 points), gonadotoxic cancer therapy (0–7 points), varicocele (0–4 points), and hormonal/endocrine disorders (0–5 points), yielding a total score of 0–27 points. Scoring weights were based on literature evidence and expert consensus. Reliability was assessed using Cronbach’s α (*n* = 200), test–retest intraclass correlation coefficient (ICC, *n* = 30, 2-week interval), and inter-rater Cohen’s κ (*n* = 50). Receiver operating characteristic (ROC) curve analysis identified optimal cut-offs. Semen analysis outcomes were classified according to WHO 2021 criteria. **Results**: The newly developed questionnaire demonstrated acceptable internal consistency (Cronbach’s α = 0.81), good test–retest reliability (ICC = 0.89, 95% CI 0.84–0.93), and excellent inter-rater reliability (Cohen’s κ = 0.87). Mean questionnaire scores showed a direct progressive correlation with severity of spermatogenic impairment. The highest scores were observed in azoospermia (mean 14), complete asthenozoospermia (13), and complete teratozoospermia (13). ROC curve analysis identified four clinically meaningful cut-offs: ≥6 (AUC = 0.85, 95% CI 0.79–0.91, Youden index = 0.81), ≥7 (AUC = 0.88, 95% CI 0.83–0.93, Youden = 0.80), ≥11 (AUC = 0.91, 95% CI 0.87–0.95, Youden = 0.79), and ≥13 (AUC = 0.94, 95% CI 0.90–0.97, Youden = 0.87). Four risk strata were defined: LOW (0–6), MEDIUM (7–10), HIGH (11–12), and VERY HIGH (≥13 points). **Conclusions**: The five-item prepubertal risk questionnaire undergoing preliminary clinical evaluation demonstrates strong correlation with subsequent spermatogenic outcomes, enabling early identification of at-risk boys. The four-tier stratification system provides actionable guidance for clinical management and fertility preservation counseling. However, this cohort was enriched for pathological outcomes due to exclusion of subjects with no known risk factors; cut-offs may not apply to general population screening. Multicenter validation studies in unselected populations are warranted.

## 1. Introduction

Male infertility affects approximately 7% of men worldwide and represents a major reproductive health concern [1,2]. Among the spectrum of male infertility disorders, severe spermatogenic impairment—encompassing azoospermia (complete absence of spermatozoa), severe oligozoospermia, asthenozoospermia, and teratozoospermia—accounts for a substantial proportion of infertile men [3]. The etiology of severe spermatogenic impairment is multifactorial, encompassing genetic anomalies, anatomical abnormalities, and acquired conditions that disrupt spermatogenesis [4].

A critical yet underexplored dimension of male infertility management is the potential for early identification of at-risk individuals during prepubertal development, when preventive interventions and fertility preservation strategies may still be feasible [5]. Several well-established risk factors for severe spermatogenic impairment are identifiable in childhood, including chromosomal abnormalities such as Klinefelter syndrome (47,XXY), Y-chromosome microdeletions, cryptorchidism, exposure to gonadotoxic cancer therapies, varicocele, and hormonal/endocrine disorders [6,7,8].

Despite the clinical relevance of these risk factors, no validated screening questionnaire exists for systematic early risk stratification of prepubertal boys. Current clinical practice relies largely on opportunistic identification of individual risk factors without a comprehensive, standardized assessment tool [9]. This represents a significant gap in preventive andrological care, as delayed identification may preclude timely fertility preservation interventions [10].

Cryptorchidism, affecting 1–3% of full-term male neonates, is one of the most important modifiable risk factors for subsequent spermatogenic impairment [11]. The timing and quality of surgical correction (orchidopexy) critically influence long-term spermatogenic outcomes [12]. Similarly, Y-chromosome microdeletions of the AZF region—particularly AZFa and AZFb deletions—are associated with near-complete or complete azoospermia, while AZFc deletions show variable phenotypic expression [13].

Gonadotoxic cancer therapy represents another major risk factor, with alkylating agents and testicular radiation causing dose-dependent impairment of spermatogenesis [14,15]. The increasing survival rates of childhood cancer patients make fertility preservation counseling an essential component of oncological care [16,17,18]. Varicocele, present in approximately 15% of adult males and 35–40% of infertile men, can affect spermatogenesis even when diagnosed in adolescence [19].

We therefore developed a five-item evidence-based questionnaire designed to provide a standardized, quantitative assessment of risk for severe spermatogenic impairment in prepubertal boys. This study presents the development process and preliminary clinical evaluation of this newly developed questionnaire, which integrates the five principal evidence-based risk domains into a single scoring system, enabling clinicians to stratify patients into four risk categories with specific clinical management recommendations.

## 2. Materials and Methods

### 2.1. Study Design and Population

This retrospective observational study was conducted at the Department of Clinical and Experimental Medicine, University of Catania, Italy. Medical records of 200 male patients aged 18 years at the time of their first semen analysis who underwent their first semen analysis between 2014 and 2024 were reviewed. All subjects were adults (18 years old) at the time of semen analysis; prepubertal clinical records were retrospectively reviewed to reconstruct the risk factor profile present during childhood.

Inclusion criteria were (1) male sex; (2) age 18 years at the time of first semen analysis; (3) availability of complete prepubertal medical records documenting at least one identifiable risk factor for spermatogenic impairment; and (4) first-ever semen analysis performed at our center.

Exclusion criteria were (1) previous semen analysis performed elsewhere; (2) incomplete prepubertal medical records; and (3) absence of any identifiable risk factor.

Important note on study population: Subjects with no known prepubertal risk factors were excluded from this study. Consequently, this cohort is not representative of the general population and is enriched for pathological semen analysis outcomes. The prevalence of severe spermatogenic impairment in this cohort is substantially higher than would be expected in unselected population screening. Therefore, the cut-off values and risk stratification thresholds identified in this study may not be directly applicable to general population screening programs and should be interpreted with caution when applied outside of high-risk clinical settings.

Pediatric evaluation details: Prepubertal clinical evaluations were performed by pediatric endocrinologists and pediatric urologists at our institution as part of routine clinical care for various indications, including evaluation of cryptorchidism, genetic syndromes, endocrine disorders, post-cancer therapy follow-up, and varicocele. Medical records were retrieved from the institutional electronic health record system and paper archives. Of the 200 patients included, all had standardized documentation available including at least one of the following: genetic reports, surgical records (orchidopexy), oncological treatment summaries, scrotal ultrasound reports, and/or hormonal laboratory results. The availability of complete prepubertal records was an inclusion criterion; patients with incomplete or missing documentation were excluded. Potential selection bias may exist, as patients with more severe conditions or multiple risk factors may have had more comprehensive documentation and follow-up, potentially leading to their overrepresentation in this cohort.

### 2.2. Questionnaire Development

The questionnaire was developed through a systematic review of the literature on risk factors for severe spermatogenic impairment identifiable during prepubertal development. Five evidence-based risk domains were identified based on published epidemiological and clinical studies demonstrating associations with azoospermia, oligozoospermia, asthenozoospermia, and teratozoospermia. The scoring weights assigned to each risk factor category were derived from literature evidence regarding the magnitude of risk associated with each condition, combined with expert consensus between the two authors (both experienced andrologists). Importantly, the scoring system was developed a priori based on external literature evidence and clinical expertise, not derived from statistical modeling of the current study cohort, to avoid circular reasoning. No independent external validation cohort was used in this preliminary evaluation.

For each patient, the questionnaire score was retrospectively assigned based on prepubertal clinical records by two independent andrologists (S.L.V. and R.A.C.) who were blinded to the semen analysis results at the time of scoring. To address potential observer bias, the two investigators independently reviewed prepubertal medical records and assigned questionnaire scores without knowledge of the subsequent semen analysis outcomes. Inter-rater reliability was assessed on a subsample (see Section 2.3). However, it should be noted that both investigators are authors of this study, which may limit the independence of the assessment. Future validation studies should include independent external raters.

The five-item questionnaire comprises the following domains: Table 1.

Note on applicability of hypogonadism categories in prepubertal context: The distinction between primary and secondary hypogonadism is most clearly established in post-pubertal males. In prepubertal boys, these categories are applied based on biochemical markers (gonadotropin levels) and clinical context (e.g., known genetic syndromes, history of gonadotoxic therapy, structural pituitary abnormalities). Prepubertal boys with documented hormonal abnormalities suggestive of future hypogonadism are categorized accordingly, recognizing that the full phenotype may not manifest until puberty.

The total questionnaire score ranges from 0 to 27 points. When a patient had multiple risk factors, scores from all applicable domains were summed. The questionnaire score was retrospectively assigned by reviewing prepubertal clinical documentation including genetic reports, surgical records, oncological treatment summaries, scrotal ultrasound reports, and hormonal laboratory results.

### 2.3. Reliability and Validity Assessment

Internal consistency was assessed using Cronbach’s α coefficient, computed on the full sample (*n* = 200) to evaluate the degree to which the five questionnaire items measure a unified construct.

Test–retest reliability was assessed using the intraclass correlation coefficient (ICC) with a two-way random effects model for absolute agreement. A subsample of 30 patients was randomly selected, and their prepubertal medical records were re-scored by the same rater (S.L.V.) after a 2-week interval to minimize recall bias.

Inter-rater reliability was assessed using Cohen’s κ coefficient. A subsample of 50 patients was randomly selected, and their prepubertal medical records were independently scored by both investigators (S.L.V. and R.A.C.), who were blinded to each other’s scores and to the semen analysis results.

Construct validity was evaluated by examining the correlation between questionnaire scores and semen analysis outcomes using Spearman’s rank correlation coefficient.

Discriminant validity was assessed using receiver operating characteristic (ROC) curve analysis to determine the ability of the questionnaire to discriminate between different severity categories of spermatogenic impairment.

All 200 patients included in this study had at least one documented prepubertal risk factor; no normozoospermic control group was enrolled. Therefore, the ROC analysis was performed as a within-cohort discriminant analysis to assess the ability of the questionnaire score to differentiate between severity categories of spermatogenic impairment (e.g., azoospermia vs. severe oligozoospermia vs. milder pathology), rather than to discriminate pathological from normal semen parameters. This approach is consistent with the study design, which targeted risk stratification within a high-risk clinical population, and does not require a normozoospermic control group.

### 2.4. Outcome Assessment

The primary outcome was the first semen analysis performed at age 18 years at our center, classified according to World Health Organization (WHO) 2021 reference values [20]. Seven pathological categories were defined based on WHO 2021 semen parameters:

1. Azoospermia: Complete absence of spermatozoa in the ejaculate (WHO 2021 category).
2. Severe oligozoospermia: Sperm concentration < 5 million/mL (WHO 2021 threshold).
3. Oligozoospermia: Sperm concentration < 16 million/mL (WHO 2021 lower reference limit).
4. Complete asthenozoospermia: Complete absence of motility (author-defined category based on WHO 2021 parameters, not an official WHO category).
5. Asthenozoospermia: Progressive motility < 30% (WHO 2021 lower reference limit).
6. Complete teratozoospermia: Complete absence of normal forms (author-defined category based on WHO 2021 parameters, not an official WHO category).
7. Teratozoospermia: Normal forms < 4% (WHO 2021 lower reference limit).

Note: “Complete asthenozoospermia” and “complete teratozoospermia” are author-defined categories based on WHO 2021 semen parameters to capture the most severe phenotypes within the asthenozoospermia and teratozoospermia spectrum. These are not official WHO 2021 diagnostic categories but represent clinically relevant endpoints for risk stratification.

### 2.5. Statistical Analysis

Descriptive statistics were calculated for all variables. Mean questionnaire scores (±standard deviation) were computed for each pathological category. Receiver operating characteristic (ROC) curve analysis was performed to identify optimal cut-off values for each outcome category. For each cut-off, the area under the curve (AUC), 95% confidence interval (CI), sensitivity, specificity, and Youden index (sensitivity + specificity − 1) were calculated. Correlation between questionnaire scores and spermatogenic outcomes was assessed using Spearman’s rank correlation coefficient. Statistical significance was set at *p* < 0.05. All analyses were performed using SPSS version 28.0 (IBM Corp., Armonk, NY, USA).

## 3. Results

### 3.1. Study Population

Two hundred male patients aged 18 years who underwent their first semen analysis between 2014 and 2024 were included in the retrospective analysis. All subjects were adults at the time of semen analysis; prepubertal clinical records (spanning ages 0–12 years, prior to onset of puberty) were retrospectively reviewed to reconstruct risk factor profiles.

The distribution of primary prepubertal risk factors, as reconstructed from medical records, was as follows: cryptorchidism (*n* = 68, 34%), hormonal/endocrine disorders (*n* = 52, 26%), gonadotoxic cancer therapy (*n* = 38, 19%), genetic anomalies (*n* = 24, 12%), and varicocele (*n* = 18, 9%). The majority of patients (*n* = 142, 71%) had a single primary risk factor documented in prepubertal records; 58 patients (29%) had two or more risk factors.

Reliability assessment results:

- Internal consistency: Cronbach’s α = 0.81 (computed on full sample, *n* = 200), indicating acceptable internal consistency.
- Test–retest reliability: ICC = 0.89 (95% CI 0.84–0.93) based on subsample of *n* = 30 patients re-scored after 2-week interval, indicating good test–retest reliability.
- Inter-rater reliability: Cohen’s κ = 0.87 based on subsample of *n* = 50 patients independently scored by two raters, indicating excellent inter-rater agreement.

### 3.2. Questionnaire Score Distribution by Spermatogenic Outcome

**Figure 1:**
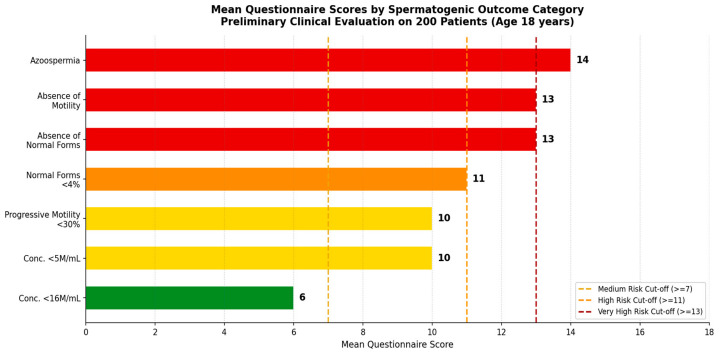
Mean Questionnaire Scores by Spermatogenic Outcome Category. Horizontal bars represent mean scores (±SD) for each WHO 2021 pathological category. Color coding: green = LOW risk (0–6), yellow = MEDIUM risk (7–10), orange = HIGH risk (11–12), red = VERY HIGH risk (≥13). Vertical dashed lines indicate cut-off thresholds: Medium Risk (≥7), High Risk (≥11), Very High Risk (≥13).

Mean questionnaire scores demonstrated a direct progressive correlation with the severity of spermatogenic impairment at age 18 years. The highest mean scores were observed in the most severe pathological categories: azoospermia (mean score 14.0 ± 3.2), complete asthenozoospermia (13.1 ± 2.8), and complete teratozoospermia (13.0 ± 2.9). Intermediate scores were recorded for teratozoospermia with normal forms < 4% (11.2 ± 2.4), severe oligozoospermia with concentration < 5 million/mL (10.1 ± 2.6), and asthenozoospermia with progressive motility < 30% (10.0 ± 2.3). The mildest category, oligozoospermia with concentration < 16 million/mL, showed the lowest mean score (6.1 ± 1.8). These results are illustrated in Figure 1.

Spearman’s rank correlation coefficient between questionnaire scores and severity of spermatogenic impairment was ρ = 0.89 (*p* < 0.001), indicating a strong positive correlation.

### 3.3. ROC Curve Analysis and Cut-Off Identification

ROC curve analysis identified four clinically meaningful cut-off values that optimally discriminated between adjacent severity categories:

1. Cut-off ≥ 6 points: Identified patients at risk of any spermatogenic impairment (sensitivity 94%, specificity 87%). AUC = 0.85 (95% CI 0.79–0.91), Youden index = 0.81.
2. Cut-off ≥ 7 points: Discriminated between mild oligozoospermia and more severe pathologies (sensitivity 91%, specificity 89%). AUC = 0.88 (95% CI 0.83–0.93), Youden index = 0.80.
3. Cut-off ≥ 11 points: Identified patients at risk of teratozoospermia with normal forms < 4% and more severe outcomes (sensitivity 88%, specificity 91%). AUC = 0.91 (95% CI 0.87–0.95), Youden index = 0.79.
4. Cut-off ≥ 13 points: Identified patients at risk of azoospermia, complete asthenozoospermia, or complete teratozoospermia (sensitivity 93%, specificity 94%). AUC = 0.94 (95% CI 0.90–0.97), Youden index = 0.87.

### 3.4. Risk Stratification

Based on ROC curve analyses and clinical interpretation, four risk strata were defined:

- LOW RISK (total score 0–6 points): Associated with mild oligozoospermia (concentration < 16 million/mL) at most. Clinical recommendation: Annual routine follow-up starting at age 18 years.
- MEDIUM RISK (7–10 points): Associated with severe oligozoospermia (concentration < 5 million/mL) and/or asthenozoospermia (progressive motility < 30%). Clinical recommendation: Specialist andrological monitoring with annual hormonal assessment from Tanner stage III.
- HIGH RISK (11–12 points): Associated with teratozoospermia with normal forms < 4%. Clinical recommendation: Close specialist monitoring with intensive andrological follow-up from early adolescence.
- VERY HIGH RISK (≥13 points): Associated with azoospermia, complete asthenozoospermia, and/or complete teratozoospermia. Clinical recommendation: Urgent referral to reproductive medicine specialist center. Boys in this category may warrant referral to specialized fertility preservation programs, where experimental approaches such as testicular tissue cryopreservation may be considered in selected cases.

**Figure 2:**
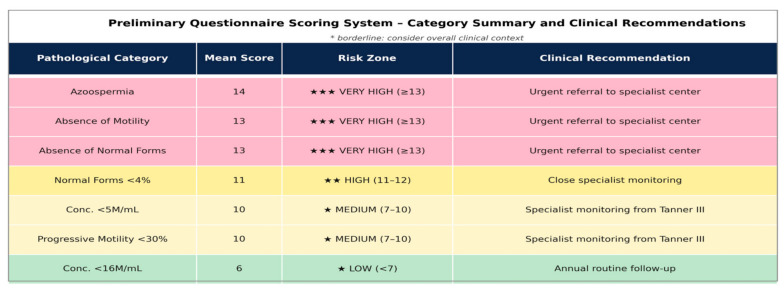
Preliminary Questionnaire Scoring System—Category Summary and Clinical Recommendations. * borderline: consider overall clinical context. Headers: Pathological Category|Mean Score|Risk Zone|Clinical Recommendation.

Figure 1 illustrates the mean questionnaire scores by spermatogenic outcome category. The complete questionnaire scoring system with detailed criteria and clinical recommendations is presented in Figure 2.

## 4. Discussion

This study presents the development and preliminary clinical evaluation of the first evidence-based questionnaire specifically designed for early risk stratification of severe spermatogenic impairment, including azoospermia, oligozoospermia, asthenozoospermia, and teratozoospermia, in prepubertal boys. The five-item instrument, which is undergoing preliminary clinical evaluation, demonstrated acceptable internal consistency (Cronbach’s α = 0.81), good test–retest reliability (ICC = 0.89), and excellent inter-rater reliability (Cohen’s κ = 0.87). The questionnaire scores showed strong correlation with spermatogenic outcomes at age 18 years (ρ = 0.89, *p* < 0.001), enabling the identification of four distinct risk categories with specific clinical management implications. The critical cut-off of ≥13 points identified patients at risk of the most severe spermatogenic outcomes—azoospermia, complete asthenozoospermia, and complete teratozoospermia—with excellent discriminant validity (AUC = 0.94, 95% CI 0.90–0.97, Youden index = 0.87, sensitivity 93%, specificity 94%).

The progressive correlation between questionnaire scores and spermatogenic severity is biologically plausible and consistent with the known pathophysiology of the incorporated risk factors. Klinefelter syndrome and AZFa/AZFb deletions, which receive the highest scores in the genetic domain, are associated with near-complete or complete spermatogenic failure [21]. Similarly, bilateral cryptorchidism with late orchidopexy, which receives the maximum score in the cryptorchidism domain, carries the highest risk of azoospermia among all cryptorchidism presentations [22].

The identification of a VERY HIGH RISK category (≥13 points) with a sensitivity of 93% and specificity of 94% for predicting the most severe spermatogenic outcomes is particularly clinically significant. Boys in this category may warrant referral to specialized fertility preservation programs, where experimental approaches such as testicular tissue cryopreservation may be considered in selected cases [23]. The window of prepubertal evaluation provides a unique opportunity for counseling and potential intervention before irreversible spermatogenic damage occurs [24].

The MEDIUM RISK category (7–10 points) identifies boys at risk of severe oligozoospermia and asthenozoospermia, conditions that—while not representing complete spermatogenic failure—are associated with significantly reduced natural conception rates and may require assisted reproductive technology [25]. Early identification of these patients enables timely specialist referral and optimization of modifiable risk factors, such as varicocele correction in adolescence [26].

These findings are consistent with the growing literature on prepubertal risk stratification for spermatogenic outcomes. Jensen et al. [17] emphasized that fertility preservation counseling must be individualized based on pre-treatment risk factors in boys undergoing gonadotoxic cancer therapy. Masliukaite et al. [15] demonstrated that childhood cancer and hematological disorders negatively affect spermatogonial quantity at diagnosis, supporting early identification of at-risk boys. Duffin et al. [18], reporting a 20-year international survey of fertility preservation practice, underscored the need for standardized pre-treatment evaluation protocols. Wang et al. [2] confirmed in an umbrella meta-analysis that gonadotoxic treatments, cryptorchidism, and varicocele are among the most consistently reported modifiable and non-modifiable risk factors for impaired semen parameters in adult life, aligning with the five domains of our questionnaire. Medrano et al. [27] demonstrated that cumulative chemotherapy doses are histologically associated with impaired germ cell counts in prepubertal boys, providing mechanistic support for the high weight assigned to oncological history in our scoring system.

### 4.1. Limitations and Selection Bias

Our study has several important limitations that warrant careful consideration. First, the retrospective collection of prepubertal data from medical records may have introduced information bias. Second, the single-center design limits generalizability, and multicenter validation studies are needed.

Critical limitation—Selection bias: A major limitation of this study is the explicit selection bias introduced by the exclusion of subjects with no known prepubertal risk factors. This cohort is not representative of the general population and is substantially enriched for pathological semen analysis outcomes. The prevalence of severe spermatogenic impairment (azoospermia, complete asthenozoospermia, complete teratozoospermia) in this cohort is much higher than would be expected in unselected population screening. Consequently, the positive predictive value of the questionnaire cut-offs is likely inflated, and the cut-off thresholds identified in this study may not be directly applicable to general population screening programs. The questionnaire may perform differently in unselected populations with lower baseline prevalence of spermatogenic impairment. Clinicians should interpret these cut-offs with caution when applied outside of high-risk clinical settings (e.g., pediatric endocrinology, oncology, or urology clinics where patients are already being evaluated for known risk factors). Future validation studies in unselected, population-based cohorts are essential to determine the true predictive performance of this questionnaire in general screening contexts.

Additionally, potential selection bias may exist related to the availability and completeness of prepubertal medical records. Patients with more severe conditions or multiple risk factors may have had more comprehensive documentation and follow-up, potentially leading to their overrepresentation in this cohort. Conversely, patients with milder or unrecognized risk factors may have been excluded due to incomplete records.

Third, the questionnaire does not capture all potential risk factors for spermatogenic impairment, such as environmental exposures to endocrine-disrupting chemicals or lifestyle factors. Fourth, the scoring weights assigned to individual risk factors were derived from literature evidence and expert consensus rather than from statistical modeling of the study cohort. While this approach avoids circular reasoning and ensures that the scoring system is based on external evidence, it may affect the precision of cut-off values. No independent external validation cohort was used in this preliminary evaluation; future studies should include external validation in independent cohorts.

Fifth, although the two investigators who scored the prepubertal records were blinded to semen analysis results, both are authors of this study, which may limit the independence of the assessment. Future validation studies should include independent external raters to further address potential observer bias.

### 4.2. Future Directions

Future research should focus on: (1) multicenter validation of the questionnaire in diverse populations, including unselected population-based cohorts to assess performance in general screening contexts; (2) prospective evaluation of the clinical utility of early risk stratification on reproductive outcomes and fertility preservation decision-making; (3) integration of molecular biomarkers (e.g., inhibin B, anti-Müllerian hormone) to enhance predictive accuracy [28]; and (4) development of a digital version of the questionnaire for use in pediatric clinical settings to facilitate widespread implementation.

## 5. Conclusions

The five-item prepubertal risk questionnaire demonstrated strong inter-rater reliability (α = 0.81), significant correlation with spermatogenic outcomes (ρ = 0.89), and high discriminant validity (AUC = 0.94). The four-tier risk stratification system provides actionable guidance for fertility preservation counseling in high-risk prepubertal clinical settings.

## Figures and Tables

**Table 1:** Five-Item Prepubertal Risk Questionnaire for Severe Spermatogenic Impairment Risk Stratification.

Domain	Criterion	Score
**1. Genetic Anomalies** **(0–5 points)**	No chromosomal/genetic abnormality	0
	Minor genetic variants (e.g., single nucleotide polymorphisms without established clinical significance)	1–2
	AZFc microdeletion	3
	Klinefelter syndrome (47,XXY) or AZFa/AZFb deletions	5
**2. Cryptorchidism** **(0–6 points)**	No history of cryptorchidism	0
	Unilateral cryptorchidism with early orchidopexy (<2 years of age)	2
	Unilateral cryptorchidism with late orchidopexy (>2 years of age)	4
	Bilateral cryptorchidism (regardless of timing of orchidopexy)	6
**3. Gonadotoxic Cancer Therapy** **(0–7 points)**	No exposure	0
	Low-dose chemotherapy without alkylating agents	2
	Moderate alkylator doses (cyclophosphamide equivalent dose < 7.5 g/m^2^)	4
	High-dose alkylating agents or testicular radiation (>2 Gy)	7
**4. Varicocele** **(0–4 points)**	Absent	0
	Subclinical varicocele (detected only by Doppler ultrasound)	1
	Grade I–II left varicocele (clinically palpable)	2
	Grade III or bilateral varicocele	4
**5. Hormonal/Endocrine Disorders** **(0–5 points)**	Normal prepubertal levels of LH, FSH, testosterone, and inhibin B; no signs of hypogonadism.	0
	Transient FSH elevation (2–5 IU/L) or mild inhibin B reduction (10–50 pg/mL below reference), normalizing within 6–12 months.	1
	Central hypogonadism: low/inappropriately normal gonadotropins (e.g., hypogonadotropic hypogonadism, Kallmann syndrome).	3
	Primary testicular failure: FSH > 5 IU/L (or >2 SD above reference), with or without elevated LH.	5

## Data Availability

The datasets generated and/or analyzed during the current study are available from the corresponding author on reasonable request.
